# SARS-CoV-2 infection and two-year longitudinal clinical trajectory in pulmonary hypertension: a single-center retrospective exploratory cohort study

**DOI:** 10.3389/fpubh.2026.1901917

**Published:** 2026-09-24

**Authors:** Arwa Alqahtani, Fatimah Alzubi, Dalia Obeid, Fatimah Alhamlan, Fatimah Alghnnam, Abdullah Al Marzan, Ahmed A. Al-Qahtani

**Affiliations:** 1 Department of Family Medicine, College of Medicine, Imam Mohammad Ibn Saud Islamic University (IMSIU), Riyadh, Saudi Arabia; 2 Department of Medicine, Pulmonary Hypertension Program, King Faisal Specialist Hospital & Research Centre, Riyadh, Saudi Arabia; 3 Organ Transplant Center of Excellence, King Faisal Specialist Hospital & Research Centre, Riyadh, Saudi Arabia; 4 Department of Infection and Immunity, King Faisal Specialist Hospital & Research Centre, Riyadh, Saudi Arabia; 5 Department of Microbiology and Immunology, College of Medicine, Alfaisal University, Riyadh, Saudi Arabia; 6 Department of Biochemistry and Molecular Biology, Shahjalal University of Science and Technology, Sylhet, Bangladesh; 7 Department of Data Science, Toxicology Society of Bangladesh, Dhaka, Bangladesh

**Keywords:** chronic thromboembolic pulmonary hypertension, COVID-19, longitudinal follow-up, pulmonary arterial hypertension, pulmonary hypertension, risk stratification, SARS-CoV-2, survival analysis

## Abstract

**Introduction:**

SARS-CoV-2 may promote endothelial injury, pulmonary vascular inflammation, and thrombosis, mechanisms relevant to pulmonary hypertension (PH), but longitudinal post-COVID-19 trajectories in established PH remain poorly defined.

**Methods:**

We retrospectively analysed 40 PH patients in the prospectively established POTENT registry with a first documented positive SARS-CoV-2 RT-PCR test dated 15 June 2020 to 2 November 2022 (index infection date). No contemporaneous non-infected PH comparator cohort was included; accordingly, the present analysis was uncontrolled and exploratory in intent. Follow-up windows were T0 during infection, T1 < 3 months, T2 3–12 months, T3 1–2 years, and T4 > 2 years. Clinical status, WHO functional class, haemodynamics, ESC/ERS risk category, NT-proBNP, and haemoglobin were evaluated. The primary endpoint was all-cause mortality, lung transplantation, ICU admission, or PH clinical deterioration, analysed using Kaplan–Meier estimation and Cox regression.

**Results:**

Median age was 37 years and 75% were female. Aetiologies were CTEPH 28%, CHD-PAH 25%, IPAH 20%, CTD-PAH 18%, and HPAH 10%; CTEPH represented WHO Group 4 PH and the others WHO Group 1 PAH. At T0, 52.5% were low risk and 27.5% intermediate-high risk. Risk profile shifted toward higher-risk categories at T2, followed by partial recovery by T4. Haemoglobin differed descriptively across available records (Kruskal–Wallis *p* = 0.03), while the patient-level exploratory mixed-effects model showed no overall time-window effect (*p* = 0.21), with all T1–T4 95% confidence intervals spanning no difference from T0. NT-proBNP was non-significant by Kruskal–Wallis (*p* = 0.3) and log10 mixed-effects modelling (*p* = 0.72). Composite event-free survival was 87.2% (95% CI 71.9–94.5%) at 1 year and 75.1% (95% CI 48.2–89.4%) at 2 years. No baseline covariate was significantly associated with the composite endpoint in the patient-level Cox analyses; for age, the univariable HR was 1.01/year (95% CI 0.95–1.07; *p* = 0.74) and the exploratory adjusted HR was 1.02 (95% CI 0.95–1.09; *p* = 0.62).

**Conclusion:**

In this single-centre retrospective exploratory cohort, SARS-CoV-2 infection was temporally associated with a post-infection worsening signal in PH trajectory, greatest at 3–12 months, with partial longer-term recovery. Findings support risk-stratified surveillance but do not establish causality or definitive COVID-19-mediated progression; controlled prospective validation is required.

## Introduction

Pulmonary hypertension (PH) is a haemodynamic syndrome characterised by elevated pulmonary arterial pressure and increased right ventricular afterload. Contemporary guidelines define PH as a resting mean pulmonary artery pressure (mPAP) > 20 mmHg, with pulmonary vascular resistance (PVR) used to distinguish pre-capillary disease phenotypes (1). The World Health Organization classifies PH into five aetiological groups, including pulmonary arterial hypertension (PAH; WHO Group 1) and chronic thromboembolic pulmonary hypertension (CTEPH; WHO Group 4) (2). Although PAH and CTEPH differ in their initiating mechanisms, both can lead to progressive elevation of PVR, right ventricular pressure overload, and clinical vulnerability to systemic stressors. This shared haemodynamic and right ventricular burden provides the rationale for evaluating post-infectious disease trajectories within a broader PH cohort, while acknowledging that the majority of the present cohort had WHO Group 1 PAH.

Patients with PH may be particularly susceptible to adverse consequences of respiratory viral infection. Even modest increases in pulmonary vascular resistance, hypoxaemia, systemic inflammation, or thrombosis can destabilise right ventricular function in patients with limited cardiopulmonary reserve. Infectious triggers have long been recognised as potential contributors to pulmonary vascular disease progression, as illustrated by HIV-associated PAH (3, 4). SARS-CoV-2 infection is clinically relevant in this context because COVID-19 can promote endothelial injury, pulmonary vascular inflammation, microvascular thrombosis, hypoxaemia, and myocardial stress, all of which may intersect with PH pathophysiology (5). Experimental and translational studies also suggest that SARS-CoV-2 spike protein signalling may activate inflammatory and pro-remodelling pathways in lung vascular cells, providing biological plausibility for pulmonary vascular effects after infection (6, 7).

Clinical data on COVID-19 in patients with established pulmonary vascular disease remain limited but concerning. Early reports from PH reference centres suggested that patients with PAH or CTEPH who developed COVID-19 experienced clinically important rates of hospitalisation and death, particularly among those with higher-risk disease (8). A national prospective cohort from the French PH network subsequently showed that COVID-19 was associated with substantial short-term mortality in patients with precapillary PH and that baseline risk status was an important determinant of outcome (9). These studies established the acute vulnerability of PH patients during SARS-CoV-2 infection, but most available evidence has focused on short-term outcomes, hospitalisation, and mortality rather than longer-term changes in disease trajectory.

From a public-health and chronic-disease-management perspective, longitudinal post-infection surveillance is needed to determine when patients with PH are most likely to experience changes in risk status, functional capacity, biomarkers, or adverse outcomes after SARS-CoV-2 infection. Registry-based follow-up can help inform risk-stratified surveillance strategies, allowing PH programmes to focus monitoring on clinically vulnerable groups rather than applying uniform follow-up intensity to all patients. This remains clinically relevant beyond the acute pandemic phase because SARS-CoV-2 continues to circulate as a recurrent respiratory infection, and patients with established PH may remain susceptible to delayed cardiopulmonary destabilisation after apparent recovery from acute infection. Accordingly, long-term observational data can support prevention-oriented follow-up and chronic disease management protocols, while remaining hypothesis-generating rather than causal.

The present study addresses this gap using data from the prospectively established POTENT registry of patients with pulmonary vascular disease treated at King Faisal Specialist Hospital and Research Centre. For the present study, we retrospectively analysed the longitudinal trajectory of 40 registry patients with confirmed SARS-CoV-2 infection, evaluating clinical status, haemodynamic and laboratory profiles, ESC/ERS risk category, and composite adverse outcomes across five pre-specified time windows from acute infection to more than two years after infection. The objective was to characterise the longitudinal disease trajectory of PH after SARS-CoV-2 infection and to identify exploratory predictors of adverse clinical outcome in this vulnerable registry-based cohort.

## Materials and methods

### Selection of patients

Pulmonary hypertension was diagnosed by right heart catheterisation (RHC) using the haemodynamic criteria applicable at the time of each patient’s original diagnosis. For the majority of the cohort, this corresponded to a resting mPAP ≥25 mmHg per the ESC/ERS 2015 guidelines (2). POTENT registry participants enrolled in the final months of the SARS-CoV-2 case-ascertainment interval may have been classified under the updated threshold of mPAP >20 mmHg with PVR > 2 Wood units per the 2022 ESC/ERS guidelines (1). However, given the small number of POTENT registry participants enrolled after August 2022, this is unlikely to have materially affected the cohort composition. Echocardiography was used as a complementary screening tool, with a peak tricuspid regurgitation velocity suggestive of elevated systolic pulmonary artery pressure (≥40 mmHg) used to guide RHC referral. Final diagnostic classification was based on the RHC-derived haemodynamic profile in all cases.

### Study design, setting, and data source

This was a single-centre retrospective longitudinal observational cohort study conducted using existing data from the POTENT registry at King Faisal Specialist Hospital and Research Centre (KFSHRC), Riyadh, Saudi Arabia. No contemporaneous non-infected PH comparator cohort was included; accordingly, the present analysis was uncontrolled and exploratory in intent. The POTENT registry itself was prospectively established as a clinical registry of patients with pulmonary vascular disease followed within the institutional Pulmonary Hypertension Program, with structured collection of clinical, haemodynamic, laboratory, treatment, and longitudinal follow-up data; however, the present SARS-CoV-2 analysis was performed retrospectively using existing registry and electronic medical-record data. The original POTENT study evaluated patients with pulmonary arterial hypertension who were switched from bosentan to macitentan (10), but the registry infrastructure also provided systematically recorded follow-up information relevant to pulmonary hypertension care. This made the registry suitable for evaluating post-COVID-19 trajectories because it included RHC-confirmed PH diagnoses, serial clinical assessments, ESC/ERS risk-related variables, haemodynamic and biomarker data, treatment history, and extended follow-up within a single tertiary referral centre.

### Eligibility criteria and cohort selection

For this retrospective analysis, all 324 POTENT registry participants were screened in registry and clinical records for documented SARS-CoV-2 infection. All documented RT-PCR-positive registry cases in the qualifying interval were retrospectively assessed for eligibility; patients were not prospectively recruited consecutively. Analytic eligibility required a first documented positive SARS-CoV-2 RT-PCR test dated 15 June 2020 to 2 November 2022; this case-ascertainment interval defines qualifying index infection dates, not original POTENT registry enrolment or the end of longitudinal follow-up. Patients also required RHC-confirmed PH based on haemodynamic assessment and clinical classification and sufficient clinical information to define T0 and subsequent follow-up windows. Patients failing the clinical eligibility criteria or lacking clinical data at the time of SARS-CoV-2 infection were excluded. Registry participants without documented SARS-CoV-2 RT-PCR positivity within this interval were not included in the infection-exposed cohort.

Among 324 registry patients screened, 47 had confirmed SARS-CoV-2 infection, 41 met the clinical inclusion criteria after record review, and one of these eligible patients was excluded because no clinical data were available at the time of infection, yielding a final analytic cohort of 40 patients. This SARS-CoV-2-positive subgroup therefore represents the infection-exposed subset of the POTENT registry during the predefined SARS-CoV-2 case-ascertainment interval rather than an unselected sample of all registry participants. Because inclusion required documented SARS-CoV-2 infection and available clinical data, the subgroup may not fully represent the broader registry population or all patients with PH treated at other centres.

### Data extraction and follow-up windows

Clinical, demographic, haemodynamic, and laboratory data were extracted from electronic medical records. The index infection date was defined as the date of the first documented positive SARS-CoV-2 RT-PCR test. This date, rather than original POTENT registry enrolment, anchored T0–T4. Symptom-onset date was not used as the primary temporal anchor because it was not uniformly available in the retrospective medical record. T0 was defined as the closest available PH-related clinical, laboratory, haemodynamic, or hospital-care assessment during the acute infection period, anchored to the index positive test date. Subsequent follow-up windows were defined relative to the index infection date as follows: T1, <3 months post-infection; T2, 3–12 months post-infection; T3, 1–2 years post-infection; and T4, >2 years post-infection. For each recorded assessment, the time from index SARS-CoV-2 RT-PCR test to assessment was calculated in days. The time from the index SARS-CoV-2 RT-PCR test to each recorded assessment was calculated in days and summarized within each follow-up window as median (IQR). The case-ascertainment interval defined index-date eligibility, not the end of longitudinal follow-up.

### Ascertainment of SARS-CoV-2 infection

SARS-CoV-2 infection was ascertained retrospectively from documented RT-PCR results in the clinical record. A qualifying case required a first documented positive test dated 15 June 2020 to 2 November 2022. Testing was performed in the context of clinical care and registry follow-up rather than as universal serial surveillance of all POTENT registry participants. For confirmed cases, RNA was extracted from nasopharyngeal swabs, followed by cDNA synthesis according to the manufacturer’s recommendations (Thermo Fisher Scientific, Hampshire, UK). SARS-CoV-2 was detected using the TaqPath COVID-19 RNase P Combo Kit 2.0 (Thermo Fisher Scientific, Hampshire, UK), which identifies three viral genes by RT-PCR, and samples showing at least two viral genes were considered positive. Accordingly, confirmed infections represent clinically documented RT-PCR-positive registry cases meeting the temporal eligibility criterion, not a population-level estimate of SARS-CoV-2 exposure across the entire POTENT registry.

### Availability of potential confounders

Potential confounders and contextual variables relevant to the post-COVID-19 trajectory were reviewed from the analysis workbook and available clinical records. Acute COVID-19 care setting was captured descriptively through hospitalization status, and PH treatment escalation was captured when documented as part of PH clinical deterioration or endpoint ascertainment. However, vaccination status, patient-level SARS-CoV-2 variant data, reinfection, concurrent respiratory infections, general comorbidity burden, and formal pre-infection PH stability were not systematically captured as structured variables in the registry analysis dataset. The availability of contextual variables relevant to residual confounding and interpretation is summarised in Supplementary Table S4.

### Statistical analysis

#### Descriptive and longitudinal analyses

Analyses were performed using R (version 4.3.0; R Foundation for Statistical Computing, Vienna, Austria), SAS (version 9.4; SAS Institute Inc., Cary, NC, United States), and GraphPad Prism 11.0.1. Continuous variables are reported as mean ± standard deviation or median (interquartile range), according to distributional properties, and categorical variables as counts and percentages. Available-record comparisons across follow-up windows used Kruskal–Wallis rank-sum tests for continuous variables and chi-square or Fisher exact tests for categorical variables, as appropriate. For longitudinal analyses, missing values were not imputed; Table 1 used available-record analysis because observations varied across windows and repeated records could occur. T0 used the closest available index-window assessment for each patient, whereas later windows could include more than one clinically recorded assessment from the same patient. For model-based analyses, T1–T4 were operationalised as <90, 90–364, 365–729, and ≥730 days after the index infection date, respectively. Unique-patient counts by window were T0 = 40, T1 = 33, T2 = 37, T3 = 33, and T4 = 7; longitudinal *p*-values were therefore interpreted as exploratory descriptive comparisons rather than definitive repeated-measures effects. As a patient-level sensitivity analysis, ESC/ERS risk distribution was reanalysed using one record per patient per window, retaining the clinically highest recorded risk category when multiple records were available.

**Table 1:** Baseline demographic, clinical, haemodynamic, laboratory, and outcome profile at T0.

Characteristic	Value
Demographic profile	
Female sex	30 (75%)
Male sex	10 (25%)
Body mass index, kg/m^2^	29 (22, 35)
Age at SARS-CoV-2 infection, years	37 (29, 46)
Pulmonary hypertension phenotype	
CHD-PAH	10 (25%)
CTD-PAH	7 (18%)
CTEPH	11 (28%)
HPAH	4 (10%)
IPAH	8 (20%)
Baseline risk and functional status	
ESC/ERS risk category: low	21 (52.5%)
ESC/ERS risk category: intermediate-low	3 (7.5%)
ESC/ERS risk category: intermediate-high	11 (27.5%)
ESC/ERS risk category: high	5 (12.5%)
WHO functional class I	14 (35%)
WHO functional class II	17 (43%)
WHO functional class III	9 (23%)
6-min walk distance, m	406 (322, 446)
Haemodynamic and laboratory profile	
Mean right atrial pressure, mmHg	10.0 (7.0, 14.0)
Mean pulmonary arterial pressure, mmHg	55 (36, 68)
Cardiac output, L/min	3.39 (2.72, 4.14)
Cardiac index, L/min/m^2^	1.92 (1.61, 2.50)
Pulmonary vascular resistance, WU	11 (6, 19)
Haemoglobin, g/L	137 (123, 144)
NT-proBNP, pg/mL	212 (78, 912)
Creatinine, μmol/L	72 (54, 87)
Care setting and recorded outcomes	
Conventional ward	2 (5.0%)
Outpatient clinic	38 (95%)
ICU admission	2 (5.0%)
Lung transplantation	1 (2.5%)
Death	4 (10%)
Cause of death among deaths (*n* = 4)	
Pulseless electrical activity arrest	1 (25%)
Right heart failure	2 (50%)
Septic shock, COVID pneumonia, and severe pulmonary hypertension	1 (25%)

Values are presented as *n* (%) or median (IQR), as appropriate. Death-cause percentages were calculated among deaths (*n* = 4).

Exploratory linear mixed-effects models were fitted for continuous outcomes with sufficient repeated observations, including haemoglobin, log10-transformed NT-proBNP, and six-minute walk distance. Each model included follow-up window as a categorical fixed effect and a patient-specific random intercept; overall time-window effects were assessed by likelihood-ratio comparison of maximum-likelihood models with and without the follow-up-window term. T0 was the reference; T1–T4 contrasts used two-sided 95% Wald confidence intervals, expressed as mean differences for haemoglobin and 6MWD and back-transformed geometric-mean ratios for log10 NT-proBNP. NT-proBNP was log10-transformed because of right-skewed distribution. Random-slope models and mixed-effects models for sparse categorical outcomes were not pursued because of the small cohort size, uneven follow-up density, limited T4 unique-patient count, and sparse category distribution. Accordingly, mixed-effects models were interpreted as exploratory sensitivity analyses, while the available-record descriptive summary was retained for contextual interpretation.

#### Time-to-event analyses and endpoint definition

Survival analyses used Kaplan–Meier estimation with log-rank testing for group comparisons and Cox proportional hazards regression for exploratory predictor analyses. Covariates were selected for clinical relevance and availability rather than by univariable significance: sex and age (demographic prognosis), 6MWD class (<406 vs. ≥ 406 m; cohort-median functional capacity), and PH aetiology (disease phenotype). Baseline ESC/ERS risk was excluded because all seven events occurred in the intermediate-high/high stratum (complete separation) and because of prognostic overlap with 6MWD. The proportional-hazards assumption was assessed in the complete-case multivariable model using covariate-specific and global Schoenfeld-residual score tests against log follow-up time. The primary composite endpoint—time to first all-cause death, lung transplantation, ICU admission, or PH clinical deterioration requiring therapy escalation—provided an overall measure of clinically consequential post-infection worsening without implying equivalent component severity. PH clinical deterioration was operationally defined as documented post-infection clinical worsening attributed to PH progression by the treating PH clinician and supported by available clinical-record evidence, including one or more of the following: escalation or initiation of advanced pulmonary vasodilator therapy, PH-related hospitalization or right-heart-failure decompensation, upward shift in WHO functional class, or clinically meaningful functional decline, including reduced six-minute walk distance when documented. Only the first chronological component counted in time-to-event analysis; later or overlapping components were reported descriptively. Endpoint ascertainment was retrospective and based on treating-clinician documentation, without blinded review, centralized endpoint adjudication, or an independent adjudication committee. Component-level events were reported separately, and robustness was assessed by excluding clinician-judged PH deterioration and retaining death, lung transplantation, and ICU admission. Seven first events informed survival analyses; the multivariable Cox model used 39 complete cases, seven events and five coefficients (≈1.4 events/coefficient), so estimates were highly exploratory and subgroup-specific Cox models were not fitted.

## Results

### Baseline demographic and clinical characteristics

Among 324 POTENT registry participants screened, 47 had a documented RT-PCR-positive index date during 15 June 2020 to 2 November 2022; 41 met the clinical inclusion criteria and one lacked clinical data at infection, yielding a final analytic cohort of 40 patients (Figure 1). These dates denote qualifying index infections, not original POTENT registry enrolment or follow-up termination. Demographic and baseline clinical characteristics are summarised in Table 1. The cohort was predominantly female (75%), with a median age of 37 years (IQR: 29–46). By PH aetiology, CTEPH was the most common diagnosis (28%), followed by CHD-PAH (25%), IPAH (20%), CTD-PAH (18%), and HPAH (10%). At T0, 52.5% were classified as low risk and 27.5% as intermediate-high risk by ESC/ERS risk stratification criteria. By WHO functional class (WHO FC), 35% were FC I, 43% were FC II, and 23% were FC III. During follow-up, four patients died, one required lung transplantation, and two were admitted to the ICU.

**Figure 1:**
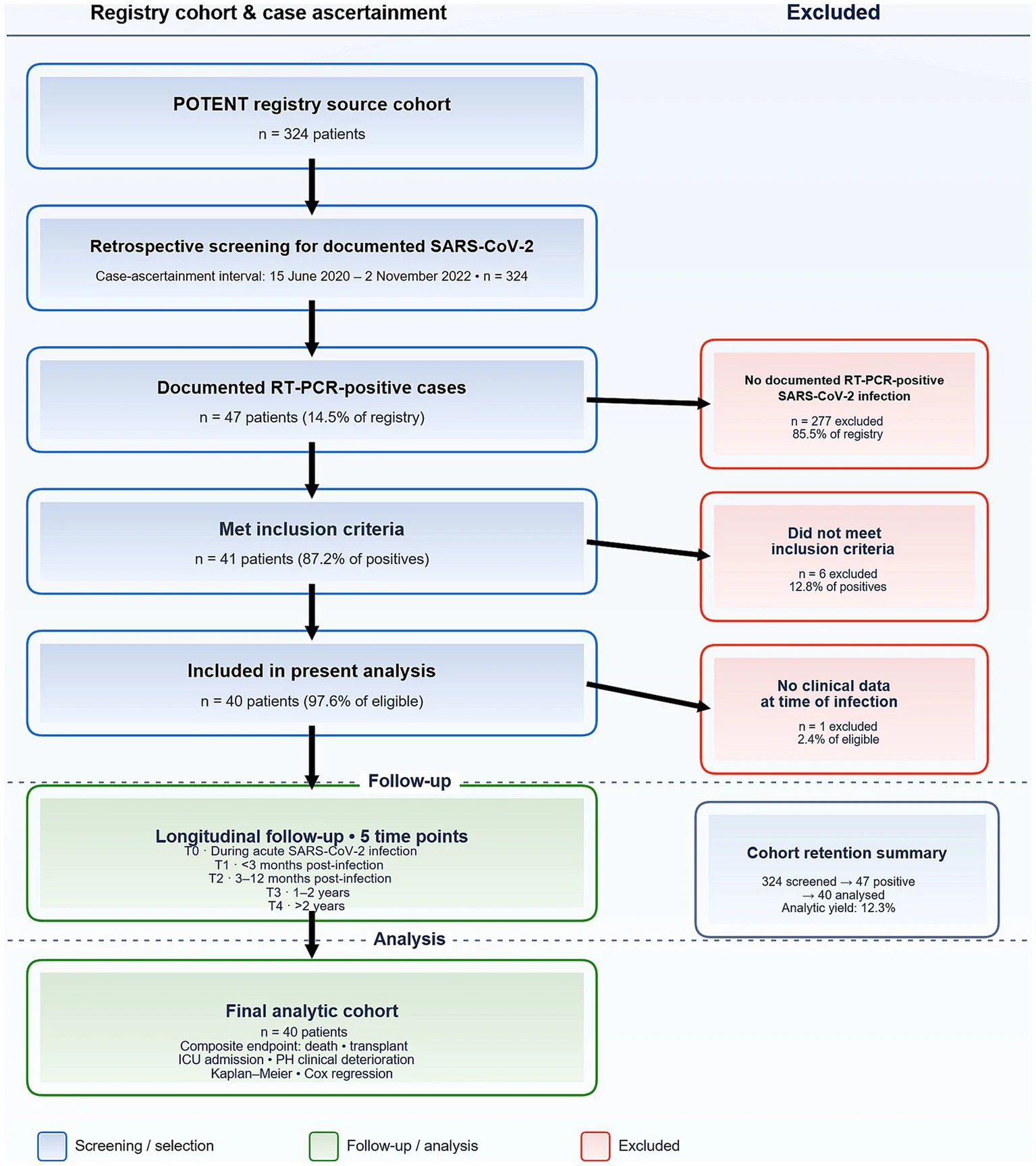
Study flow diagram. Retrospective case ascertainment and cohort selection from the POTENT registry, showing the 15 June 2020 to 2 November 2022 index-date interval, documented RT-PCR-positive cases, exclusions, and T0–T4 follow-up relative to the index infection date.

### Clinical data during and after COVID-19 infection

Clinical and demographic data stratified by visit time point are presented in Table 2 and Figure 2. Table 2 summarises available records within each follow-up window; the corresponding unique-patient counts were 40 at T0, 33 at T1, 37 at T2, 33 at T3, and 7 at T4. Risk stratification category was significantly associated with time point in the available-record analysis (*p* < 0.001). The proportion of records classified as intermediate-high or high risk increased from 40.0% at T0 to 58.2% at T2 (three to twelve months post-infection), consistent with a period of maximal disease impact. By T4, the high-risk and intermediate-high-risk categories had resolved entirely among available records, with 58% classified as intermediate-low and 42% as low risk.

**Table 2:** Longitudinal clinical, haemodynamic, and laboratory trajectory across follow-up windows.

Characteristic	T0 During infection	T1 < 3 months	T2 3–12 months	T3 1–2 years	T4 > 2 years	*p* value
Assessment timing						
Time from index SARS-CoV-2 RT-PCR test to recorded assessment, days, median (IQR)	7 (2–14)	56 (42–75)	180 (120–270)	548 (450–640)	850 (780–930)	—
ESC/ERS risk assessment						
Available risk-assessment records, n	40	42	79	65	19	
High	5 (12.50%)	6 (14.29%)	20 (25.32%)	12 (18.46%)	0 (0.00%)	*p* < 0.001
Intermediate-low	3 (7.50%)	7 (16.67%)	14 (17.72%)	13 (20.00%)	11 (57.89%)	
Intermediate-high	11 (27.50%)	10 (23.81%)	26 (32.91%)	10 (15.38%)	0 (0.00%)	
Low	21 (52.50%)	19 (45.24%)	19 (24.05%)	30 (46.15%)	8 (42.11%)	
Hospitalization setting						
Available records, *n*	40	45	75	66	19	
Conventional ward	2 (5.00%)	4 (8.89%)	9 (12.00%)	8 (12.12%)	1 (5.26%)	0.46
Intensive care unit	0 (0.00%)	2 (4.44%)	1 (1.33%)	0 (0.00%)	0 (0.00%)	
Outpatient clinic	38 (95.00%)	39 (86.67%)	65 (86.67%)	58 (87.88%)	18 (94.74%)	
Hospitalization attributed to PH deterioration						
Available records, *n*	40	45	75	66	19	
No	38 (95.00%)	44 (97.78%)	73 (97.33%)	65 (98.48%)	19 (100.00%)	0.88
Yes	2 (5.00%)	1 (2.22%)	2 (2.67%)	1 (1.52%)	0 (0.00%)	
Haemodynamic profile						
Mean right atrial pressure, mmHg	10 (7, 14)	17 (17, 17)	18 (11, 23)	12 (10, 20)	14 (13, 14)	0.30
Mean pulmonary arterial pressure, mmHg	55 (36, 68)	34 (34, 34)	54 (39, 59)	58 (40, 73)	57 (56, 57)	0.90
Cardiac output, L/min	3.39 (2.72, 4.14)	Not available	3.01 (2.67, 3.20)	3.86 (3.78, 3.93)	4.30 (4.16, 4.43)	0.14
Cardiac index, L/min/m^2^	1.92 (1.61, 2.50)	Not available	1.70 (1.63, 1.78)	2.10 (1.90, 2.30)	2.35 (2.28, 2.43)	0.30
Pulmonary vascular resistance, WU	11 (6, 19)	Not available	9 (8, 16)	14 (14, 14)	10 (9, 10)	>0.90
Laboratory profile						
Haemoglobin, g/L	137 (123, 144)	126 (108, 144)	128 (107, 148)	136 (122, 150)	149 (132, 165)	0.03
NT-proBNP, pg/mL	212 (78, 912)	146 (79, 1,225)	209 (79, 1,507)	149 (95, 757)	100 (81, 159)	0.30
Creatinine, μmol/L	72 (54, 87)	74 (59, 85)	71 (57, 85)	72 (55, 89)	58 (53, 73)	0.60

Values are presented as *n* (%) or median (IQR), as appropriate. The index infection date was defined as the date of the first documented positive SARS-CoV-2 RT-PCR test. T0 denotes the closest available PH-related clinical, laboratory, haemodynamic, or hospital-care assessment during the acute infection period; T1, <3 months post-infection; T2, 3–12 months post-infection; T3, 1–2 years post-infection; and T4, >2 years post-infection. The assessment-timing row reports the time from index SARS-CoV-2 RT-PCR test to recorded assessment within each follow-up window, expressed as median (IQR) days. Percentages for categorical variables were calculated using the available records within each section. Available-record denominators do not necessarily represent unique-patient counts, because patients could contribute more than one clinically recorded assessment within a follow-up window. The corresponding unique-patient counts by follow-up window were T0 = 40, T1 = 33, T2 = 37, T3 = 33, and T4 = 7. Therefore, this table should be interpreted as a descriptive available-record summary rather than a strict one-record-per-patient longitudinal analysis. *p* values were calculated using Fisher’s exact test or the chi-square test for categorical variables and the Kruskal–Wallis rank-sum test for continuous variables, as appropriate.

**Figure 2:**
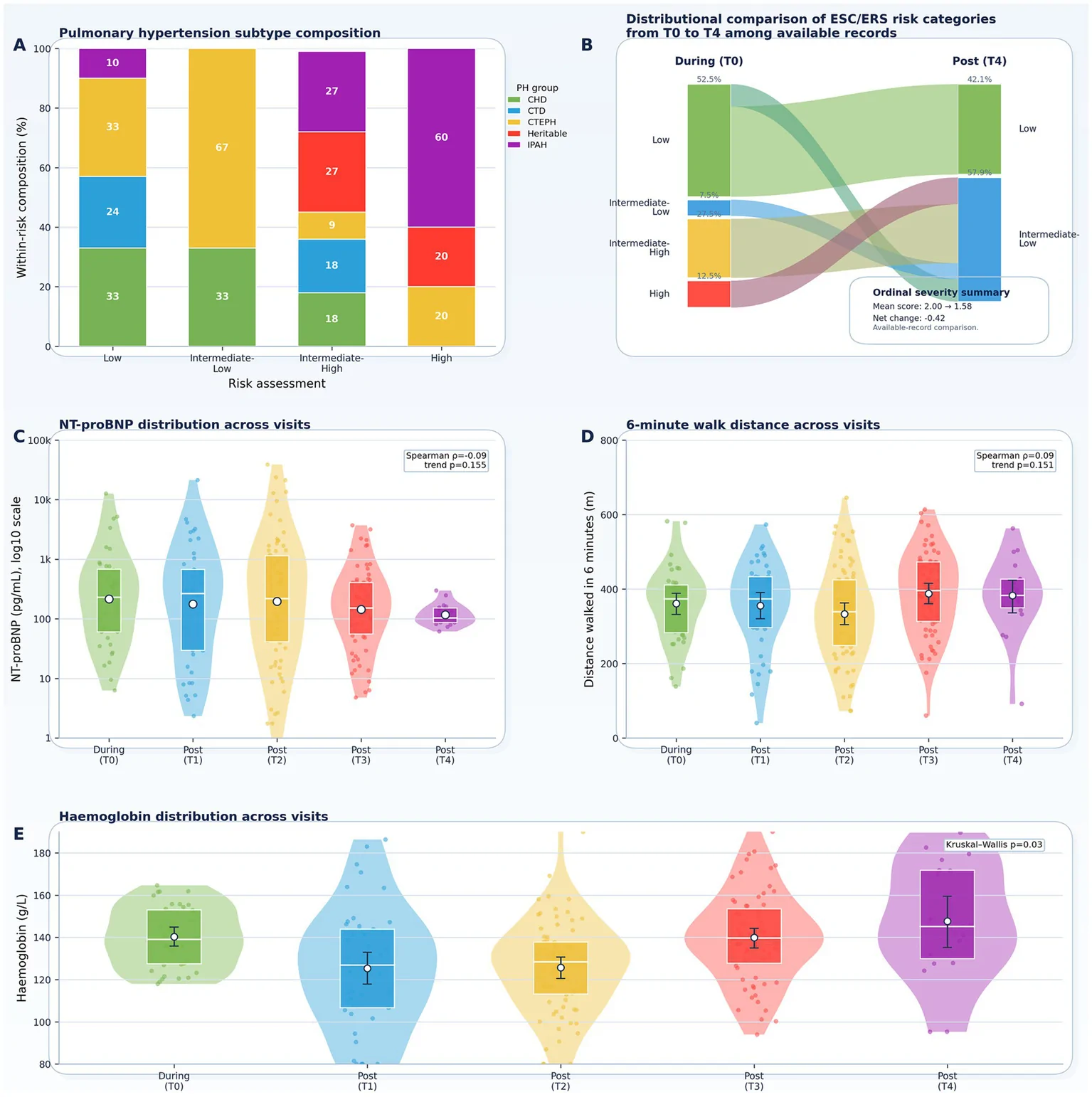
Longitudinal clinical and biomarker trajectories after SARS-CoV-2 infection. **(A)** Pulmonary hypertension aetiology distribution across baseline risk categories. **(B)** Distributional comparison of ESC/ERS risk categories between T0 and T4 among available records; this panel does not represent a paired patient-level transition analysis. **(C)** NT-proBNP distribution across visits. **(D)** Six-minute walk distance distribution across visits. **(E)** Haemoglobin distribution across visits. Panels C–E are descriptive visual summaries of available records; formal statistical comparisons and exploratory mixed-effects analyses are reported in the Results.

To address the possibility that patients with more frequent follow-up could be overrepresented in the available-record analysis, a sensitivity analysis using one ESC/ERS risk record per patient per follow-up window showed the same qualitative pattern, with the highest intermediate-high/high-risk burden during T2 and resolution by T4 among patients with available longer-term follow-up (Supplementary Table S1). In this patient-level sensitivity analysis, the proportion classified as intermediate-high or high risk was 40.0% at T0, 42.4% at T1, 48.6% at T2, 36.4% at T3, and 0.0% at T4. This supported the same qualitative pattern of greatest post-acute risk burden during the T2 window and recovery among patients with available longer-term follow-up, although the magnitude of the T2 shift was attenuated compared with the available-record analysis. Only two patients required hospitalisation during acute COVID-19 infection. The 6MWD trajectory is visualised in Figure 2D and did not show a sustained post-infection decline. In available-record descriptive comparisons, haemoglobin concentration differed across time points (Kruskal–Wallis *p* = 0.03), reaching its lowest median value at T1 (126 g/L, IQR 108–144 g/L) and subsequently recovering above the T0 value by T4 (149 g/L, IQR 132–165 g/L; Figure 2E). In the patient-level exploratory linear mixed-effects model, the overall time-window effect for haemoglobin was not statistically significant (*p* = 0.21); estimated mean changes versus T0 were −3.8 g/L (95% CI − 10.1 to 2.4) at T1, −4.3 (−10.0 to 1.4) at T2, −1.5 (−7.4 to 4.4) at T3, and +4.6 (−4.3 to 13.6) at T4. NT-proBNP remained non-significant in the available-record comparison (Kruskal–Wallis *p* = 0.3) and in the patient-level log10 mixed-effects model (overall *p* = 0.72); back-transformed geometric-mean ratios versus T0 were 1.22 (95% CI 0.93–1.61), 1.06 (0.83–1.37), 1.07 (0.82–1.39), and 1.09 (0.73–1.62) at T1–T4, respectively. For 6MWD, the patient-level mixed-effects model also showed no overall time-window effect (*p* = 0.67); estimated mean changes versus T0 were −9 m (95% CI − 39 to 21), −5 m (−29 to 20), +6 m (−19 to 32), and +17 m (−21 to 55) at T1–T4, respectively. Full model estimates and 95% confidence intervals are reported in Supplementary Table S3. These findings support interpretation of the longitudinal biomarker and functional trajectories as descriptive and hypothesis-generating rather than definitive patient-level repeated-measures effects.

### Survival analysis and risk of clinical deterioration

Survival analysis was performed using the composite endpoint of all-cause death, lung transplantation, ICU admission, and PH clinical deterioration. Seven first events occurred: PH clinical deterioration in 3/7 (42.9%), ICU admission in 2/7 (28.6%), and all-cause death in 2/7 (28.6%). The single lung transplantation occurred after an earlier PH deterioration event and was therefore not a first event. Across follow-up, four deaths, two ICU admissions, and one lung transplantation occurred, with overlap between components. Patient-level first-event timing and overlapping or subsequent components are reported in Supplementary Table S2.

Kaplan–Meier estimation demonstrated overall composite event-free survival of 87.2% (95% CI 71.9–94.5%) at 1 year and 75.1% (95% CI 48.2–89.4%) at 2 years, as shown in Figure 3A. Exploratory baseline-risk stratification showed separation in event-free survival (Figure 3B): low/intermediate-low patients remained event-free during observed follow-up, whereas events occurred in the high/intermediate-high stratum. Because only seven first events occurred in small strata, this comparison is hypothesis-generating rather than definitive subgroup inference. The objective-component sensitivity analysis (five events) showed the same qualitative pattern but remained sparse.

**Figure 3:**
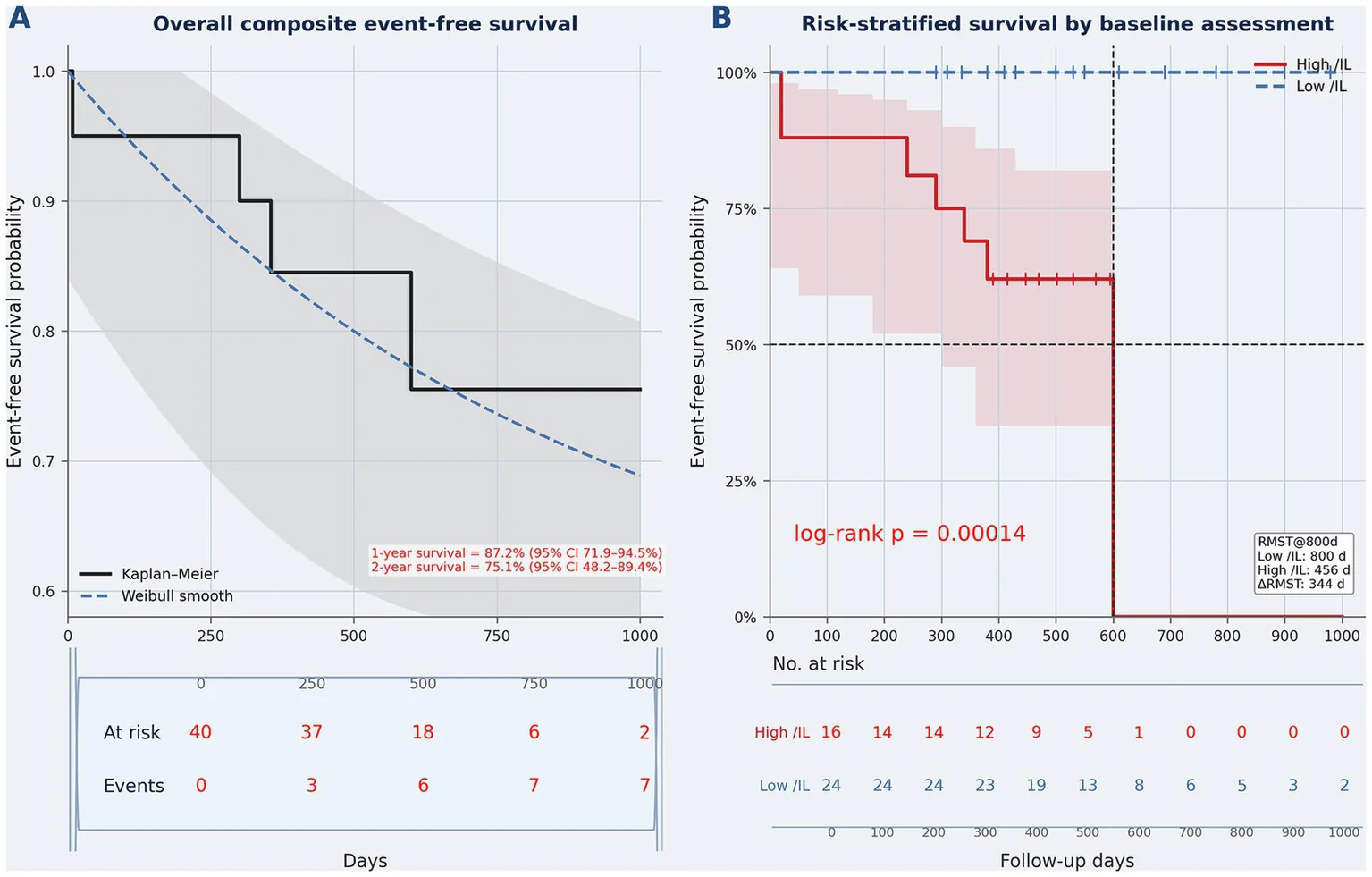
Composite event-free survival after SARS-CoV-2 infection. **(A)** Overall Kaplan–Meier event-free survival curve with fitted Weibull reference curve shown for visual orientation. **(B)** Event-free survival stratified by baseline ESC/ERS risk category, with number-at-risk table. Shaded bands show pointwise 95% confidence intervals.

An exploratory log-rank comparison between baseline risk strata was significant (*p* = 0.00014), but sparse events limit subgroup inference. Cox regression results are presented in Table 3. The patient-level Cox analyses did not identify a statistically significant baseline predictor. In univariable models, age had HR = 1.01/year (95% CI 0.95–1.07; *p* = 0.74), while the exploratory adjusted estimate was HR = 1.02 (95% CI 0.95–1.09; *p* = 0.62); estimates for sex, 6MWD class, and PH aetiology were also non-significant. The multivariable model used 39 complete cases and fitted five predictor coefficients with seven events (≈1.4 events/coefficient), so adjusted estimates remain highly unstable and vulnerable to overfitting. Schoenfeld-residual score tests against log follow-up time showed no statistically significant evidence of non-proportional hazards for sex (*p* = 0.36), age (*p* = 0.085), 6MWD (*p* = 0.20), or PH aetiology (2 df; *p* = 0.19), and the global test was non-significant (5 df; *p* = 0.12). Given only seven events, these diagnostics have limited power and non-significance should not be interpreted as proof of proportionality. Non-significant Cox estimates likewise do not establish absence of association. These results are hypothesis-generating.

**Table 3:** Exploratory Cox proportional hazards models for composite adverse outcome.

Characteristic	Univariable HR (95% CI)	*p* value	Multivariable HR (95% CI)	*p* value
Sex				
Male	Reference	-	Reference	-
Female	2.25 (0.27–18.75)	0.45	1.64 (0.17–16.33)	0.67
Age at SARS-CoV-2 infection				
Per 1-year increase	1.01 (0.95–1.07)	0.74	1.02 (0.95–1.09)	0.62
6-min walk distance class				
6MWD ≥ 406 m	Reference	-	Reference	-
6MWD < 406 m	2.58 (0.50–13.30)	0.26	2.57 (0.44–14.91)	0.29
Pulmonary hypertension aetiology				
Systemic/idiopathic	Reference	-	Reference	-
Genetic/developmental	0.83 (0.17–4.20)	0.82	1.03 (0.17–6.09)	0.97
Thromboembolic	0.36 (0.04–3.51)	0.38	0.29 (0.03–3.14)	0.31

HR denotes hazard ratio and CI denotes confidence interval. The composite adverse outcome was analysed as time to first qualifying event and was defined as all-cause death, lung transplantation, ICU admission, or PH clinical deterioration requiring escalation of therapy. Patients with overlapping components contributed only the first chronological event to the Cox model. Univariable models used 40 patients except the 6MWD model (*n* = 39); the multivariable model used 39 complete cases because baseline 6MWD was unavailable for one non-event patient, with seven events and five fitted predictor coefficients. Covariates were selected for clinical relevance and availability rather than univariable significance. Proportional hazards were assessed using Schoenfeld-residual score tests against log-transformed follow-up time; the global test was non-significant (*p* = 0.12). Cox regression estimates are exploratory because of the sparse event count and potential overfitting.

## Discussion

This retrospective longitudinal study characterises the clinical trajectory of patients with PH following SARS-CoV-2 infection over a follow-up period exceeding two years. The principal findings are as follows: (i) risk stratification worsened significantly during the three-to-twelve-month post-infection window (T2), with recovery by T4 among patients with available follow-up; (ii) haemoglobin concentration declined during the early post-acute phase before recovering above baseline; (iii) NT-proBNP fluctuated non-monotonically without a statistically significant omnibus difference across time points; and (iv) the exploratory Cox analysis did not identify a statistically significant baseline predictor, and multivariable inference remained limited by the sparse event count. These findings are broadly consistent with, but extend, the limited existing literature on COVID-19 in PH. Belge and colleagues reported, in a European survey of PH centres, that COVID-19 was associated with PH-related hospitalisation and death, with high-risk patients experiencing disproportionately worse outcomes (8). Similarly, Montani and colleagues, in a national prospective cohort study of the French PH network, demonstrated that COVID-19 was associated with substantial short-term mortality in precapillary PH patients, with in-hospital mortality substantially exceeding that of the general population, and that higher baseline risk category independently predicted adverse outcomes (9). The same cohort showed impaired functional status and elevated biomarkers in those with more severe COVID-19 presentations, broadly consistent with the risk stratification trajectory observed in the present study (9).

The current clinical relevance of these findings lies in post-acute risk management rather than acute pandemic triage. The observed peak in risk burden during the 3–12-month window supports continued clinical vigilance after SARS-CoV-2 infection, particularly among PH patients with higher baseline ESC/ERS risk. At a programme level, these data support risk-stratified long-term surveillance pathways that integrate post-infection follow-up into routine PH care, while avoiding overinterpretation of the findings as proof of causal COVID-19-related progression.

The present study adds longitudinal granularity by describing disease parameters across five follow-up windows over more than two years. The greatest observed risk burden occurred during T2 (three to twelve months post-infection), followed by lower-risk categories among patients with available longer-term follow-up. This non-monotonic temporal pattern may be compatible with a transient post-infection perturbation; however, in the absence of a contemporaneous non-infected PH comparator cohort, it cannot distinguish an effect of SARS-CoV-2 from the underlying course of PH, treatment changes, differential follow-up, or other time-varying factors. The temporal pattern should therefore be interpreted as descriptive and hypothesis-generating rather than as evidence of a causal SARS-CoV-2 effect. Studies in the general population have documented elevated cardiovascular risk persisting beyond the acute infection phase (11), and genomic analyses have identified shared molecular pathways between SARS-CoV-2 infection and cardiovascular disease (12), lending further biological plausibility to the observed trajectory. This interpretation is also compatible with established PH pathobiology, in which abnormalities of membrane-receptor signalling, ion-channel function, calcium homeostasis, and pulmonary vascular remodelling contribute to disease progression (32).

Age is an established prognostic factor in PAH and adverse COVID-19 outcomes in prior cohorts (13–19), but the patient-level Cox analysis did not detect an association with the present composite endpoint (univariable HR = 1.01 per year, 95% CI 0.95–1.07, *p* = 0.74; exploratory adjusted HR = 1.02, 95% CI 0.95–1.09, *p* = 0.62). No other Cox covariate reached statistical significance. This lack of detectable association should not be interpreted as evidence of no prognostic effect: only seven composite events were available, the adjusted model used 39 complete cases and five fitted predictor coefficients (≈1.4 events/coefficient), and confidence intervals were wide. Schoenfeld-residual diagnostics against log follow-up time did not identify a statistically significant proportional-hazards violation (global *p* = 0.12), but the diagnostic itself was event-limited. Accordingly, the Cox analyses support no independent prognostic claim for age, sex, 6MWD, or PH aetiology. The mixed-aetiology cohort was also too small for reliable subgroup-specific inference, including a separate WHO Group 1 analysis; subgroup observations are descriptive only and require validation in substantially larger cohorts.

Haemoglobin was the only laboratory parameter that differed significantly across follow-up windows in the primary available-record descriptive analysis; however, this overall time-window effect was not statistically significant in exploratory linear mixed-effects modelling that accounted for within-patient correlation. Median haemoglobin declined during the early post-acute period and subsequently recovered by longer-term follow-up. This observation is biologically plausible, as SARS-CoV-2 infection has been associated with altered haemoglobin–oxygen affinity, inflammatory effects on erythropoiesis, and impaired oxygen homeostasis (20, 21). These mechanisms may be particularly relevant in PH, where oxygen delivery can be affected by ventilation–perfusion mismatch, reduced diffusing capacity, and limited cardiopulmonary reserve (22). However, the present analysis was exploratory, based on available records, and not designed to establish patient-level repeated-measures effects or causal relationships. The haemoglobin trajectory should therefore be interpreted as a descriptive post-infection finding rather than evidence that anaemia mediated PH worsening. Recovery of haemoglobin by T4 may be consistent with post-infectious haematological recovery, although its clinical significance in this cohort remains uncertain (21). Altered haemoglobin–oxygen affinity has also been described in other chronic hypoxic or respiratory conditions, providing supportive physiological context but not direct mechanistic evidence for the pattern observed here (23, 24).

Acute respiratory distress syndrome (ARDS) is a recognised complication of severe COVID-19, and the associated haemodynamic stress may be reflected in NT-proBNP dynamics. NT-proBNP is released from cardiac myocytes in response to ventricular wall stress and mechanical load (25), and is a validated prognostic biomarker in PAH, correlating with disease severity, functional class, and haemodynamic deterioration (25–27). Its prognostic value is additionally well-established in COVID-19, where elevated NT-proBNP concentrations are independently associated with in-hospital mortality and adverse cardiovascular outcomes (28–30). Dong and colleagues reported myocardial injury in patients with end-stage heart failure during COVID-19, with NT-proBNP elevation correlated with disease progression and mortality (31). The prognostic relevance of NT-proBNP during SARS-CoV-2 infection has also been demonstrated in patients with chronic heart failure, further supporting its role as a marker of cardiovascular stress in this setting (33). In the present cohort, NT-proBNP medians fluctuated across time points and were 212 pg/mL at T0, 146 pg/mL at T1, 209 pg/mL at T2, 149 pg/mL at T3, and 100 pg/mL at T4. The overall Kruskal–Wallis test across all five time points did not reach statistical significance (*p* = 0.3). Therefore, these data do not support a definitive longitudinal increase in NT-proBNP after SARS-CoV-2 infection, although individual-level variability may still reflect transient right ventricular wall stress in selected patients. Taken together, the NT-proBNP findings should be interpreted as descriptive and hypothesis-generating. The potential utility of serial NT-proBNP monitoring in guiding post-COVID-19 follow-up intensity in PH patients warrants prospective evaluation in larger cohorts with patient-level longitudinal modelling.

These findings should also be interpreted within the broader prognostic framework of PH. Registry-based risk analyses and burden-of-disease studies demonstrate the importance of baseline disease severity, functional status, and overall disease burden when interpreting subsequent clinical outcomes (34, 35). Endothelial dysfunction and proliferative pulmonary vascular remodelling are central components of PAH pathogenesis and may contribute to heterogeneity in disease trajectory (36, 37). Long-term cohort and registry studies further demonstrate substantial variation in survival across PAH and PH populations and treatment contexts (38–41). In addition, adaptive versus maladaptive right-ventricular remodelling is an important determinant of clinical progression, while concomitant left-heart disease may further influence PH phenotype and outcome (42, 43).

Several limitations should be considered. First, this was a descriptive and hypothesis-generating study in which the composite endpoint included a clinician-judged PH deterioration component ascertained retrospectively from treating-clinician documentation rather than through blinded review or centralized independent adjudication. Although PH deterioration was supported by objective clinical-record indicators, including therapy escalation, PH-related hospitalization, right-heart-failure decompensation, WHO functional class worsening, or functional decline when documented, subjective ascertainment bias cannot be excluded. To improve transparency, component-level first events and an objective-component sensitivity analysis are reported; because the components differ in clinical severity, the aggregate composite should be interpreted alongside the component-level results.

Second, the cohort of 40 patients and seven events limited statistical power and model stability. The multivariable Cox model used 39 complete cases because baseline 6MWD was unavailable for one non-event patient; five fitted coefficients yielded approximately 1.4 events per coefficient, creating substantial overfitting risk, so adjusted estimates are exploratory. Although Schoenfeld-residual tests against log follow-up time did not detect a significant proportional-hazards violation (global *p* = 0.12), only seven events contributed to the diagnostic and its power was limited. The cohort was also insufficient for reliable subgroup inference, so aetiology- and WHO-group-specific observations are descriptive. Longitudinal analyses were further limited by uneven follow-up density, sparse longer-term observations, and potential informative missingness, especially at T4. These issues were addressed by reporting unique-patient counts by window, adding a one-record-per-patient sensitivity analysis for ESC/ERS risk category, and interpreting longitudinal comparisons as exploratory. Exploratory linear mixed-effects models were added for key continuous outcomes, but these models remained limited by small sample size, unbalanced follow-up, sparse T4 observations, and retrospective data capture; therefore, both available-record comparisons and mixed-effects analyses should be interpreted as descriptive and hypothesis-generating rather than confirmatory longitudinal inference. The model-based 95% confidence intervals for longitudinal T1–T4 contrasts were wide and included the null for haemoglobin, NT-proBNP, and 6MWD, reinforcing the descriptive and hypothesis-generating interpretation.

Third, residual confounding and selection effects cannot be excluded. The POTENT registry represents a treatment-refractory PH population with prior suboptimal response to bosentan and escalation to macitentan; therefore, absolute event rates and biomarker patterns may not represent newly diagnosed or treatment-naive PH patients. In addition, the SARS-CoV-2-positive cohort represented the infection-exposed subset of the registry rather than a randomly selected sample of all registry participants. Consistent with the RT-PCR-based ascertainment strategy, the observed 14.5% confirmed infection proportion reflects clinically documented RT-PCR-positive registry cases during the predefined SARS-CoV-2 case-ascertainment interval rather than a population-level estimate of SARS-CoV-2 exposure across the entire POTENT registry. Because testing was not universal serial surveillance, complete infection capture cannot be assured. Acute COVID-19 care setting was available descriptively, and PH treatment escalation was captured when documented as part of PH deterioration or endpoint ascertainment; however, vaccination status, patient-level SARS-CoV-2 variant data, reinfection, concurrent respiratory infections, general comorbidity burden, and formal pre-infection PH stability were not systematically available as structured variables. These contextual data constraints are summarised in Supplementary Table S4 and should be considered when interpreting the observed post-COVID-19 trajectory.

Fourth, the present study was retrospective and uncontrolled; specifically, no contemporaneous non-infected PH comparator cohort was included. This design prevents direct attribution of the observed trajectory to SARS-CoV-2 infection itself. The temporal pattern of worsening followed by recovery provides descriptive temporal context but does not establish causality. Most patients were managed as outpatients during acute infection, so the findings predominantly reflect mild-to-moderate clinically documented COVID-19 and may not generalise to severe or critical infection requiring hospitalisation. The SARS-CoV-2 ascertainment period from June 2020 to November 2022 also spanned pre-vaccination and post-vaccination phases, and vaccination against SARS-CoV-2 was introduced in Saudi Arabia in late 2020; unmeasured vaccination and variant-period effects may therefore have influenced the observed trajectory. Finally, this was a single-centre study at a tertiary referral centre in Saudi Arabia, which may limit generalisability to other healthcare settings, PH populations, treatment backgrounds, or variant periods. Multicentre prospective studies with structured covariate capture and patient-level longitudinal modelling are needed to validate these observations. Reporting of this observational cohort study was assessed against the STROBE recommendations; the completed reporting checklist is provided in Supplementary Table S5.

## Conclusion

This single-centre retrospective exploratory cohort identified an observational signal of temporally associated worsening in PH trajectory after SARS-CoV-2 infection, with the greatest observed risk burden during the 3–12-month post-infection window and partial recovery during longer-term follow-up. These findings should be interpreted as hypothesis-generating evidence supporting risk-stratified post-infection surveillance and careful chronic disease management in PH, particularly among those with higher baseline risk; the exploratory Cox analysis did not establish any independent baseline prognostic predictor. The findings do not constitute conclusive evidence of permanent or causal COVID-19-mediated PH deterioration. Because causal inference is limited by the absence of a contemporaneous non-infected PH comparator cohort, small sample size, incomplete confounder capture, and the exploratory nature of the analyses, future validation should be pursued in adequately powered, multicentre, matched controlled, prospective longitudinal cohorts with structured covariate capture, adjudicated outcomes, and patient-level longitudinal modelling.

## Data Availability

The original contributions presented in the study are included in the article/Supplementary material, further inquiries can be directed to the corresponding author.

## Glossary

ARDS: Acute Respiratory Distress Syndrome
CC: Cardiac Catheterization
COVID-19: Coronavirus Disease 2019
ECC: Echocardiography
HR: Hazard Ratio Estimate
IQR: Interquartile Range
NT-proBNP: N-terminal Pro-brain Natriuretic Peptide
NYHA/WHO FC: New York Heart Association/World Health Organization Functional Class
PAH: Pulmonary Arterial Hypertension
PH: Pulmonary Hypertension
SARS-CoV-2: Severe Acute Respiratory Syndrome Coronavirus 2
SD: Standard Deviation
WHO: World Health Organization
6MWD: Six-Minute Walk Distance
CTEPH: Chronic Thromboembolic Pulmonary Hypertension
EPV: Events Per Variable
KM: Kaplan–Meier
mPAP: Mean Pulmonary Artery Pressure
PVR: Pulmonary Vascular Resistance
RHC: Right Heart Catheterisation
RV: Right Ventricle / Right Ventricular
LMM: Linear Mixed-Effects Model
