# Supplementary material for: SARS-CoV-2 infection and two-year longitudinal clinical trajectory in pulmonary hypertension: a single-center retrospective exploratory cohort study
Source: Front Public Health. 2026 Sep 24;14:1901917. doi: 10.3389/fpubh.2026.1901917 (PMC13646160; doi:10.3389/fpubh.2026.1901917)
Supplement: Supplementary file 1 [file Table_1.docx]

***Supplementary Table S1.*** *Patient-level sensitivity analysis of ESC/ERS risk category using one record per patient per follow-up window*

| **Follow-up window** | **Unique patients** | **Low** | **Intermediate-low** | **Intermediate-high** | **High** | **Intermediate-high/high** |
| --- | --- | --- | --- | --- | --- | --- |
| T0 | 40 | 21 (52.5%) | 3 (7.5%) | 11 (27.5%) | 5 (12.5%) | 16 (40.0%) |
| T1 | 33 | 14 (42.4%) | 5 (15.2%) | 7 (21.2%) | 7 (21.2%) | 14 (42.4%) |
| T2 | 37 | 14 (37.8%) | 5 (13.5%) | 11 (29.7%) | 7 (18.9%) | 18 (48.6%) |
| T3 | 33 | 15 (45.5%) | 6 (18.2%) | 6 (18.2%) | 6 (18.2%) | 12 (36.4%) |
| T4 | 7 | 3 (42.9%) | 4 (57.1%) | 0 (0.0%) | 0 (0.0%) | 0 (0.0%) |

*Values are presented as n (%) unless otherwise indicated. T0 denotes the closest available PH-related clinical, laboratory, haemodynamic, or hospital-care assessment during the acute infection period; T1, <3 months post-infection; T2, 3-12 months post-infection; T3, 1-2 years post-infection; and T4, >2 years post-infection. For this sensitivity analysis, one ESC/ERS risk record was retained per patient per follow-up window; when more than one risk record was available within a window, the clinically highest ESC/ERS risk category recorded in that window was retained, ranked as low < intermediate-low < intermediate-high < high. Percentages were calculated using the number of unique patients contributing data within each follow-up window.*
