# Supplementary material for: SARS-CoV-2 infection and two-year longitudinal clinical trajectory in pulmonary hypertension: a single-center retrospective exploratory cohort study
Source: Front Public Health. 2026 Sep 24;14:1901917. doi: 10.3389/fpubh.2026.1901917 (PMC13646160; doi:10.3389/fpubh.2026.1901917)
Supplement: Supplementary file 2 [file Table_2.docx]

***Supplementary Table S2.*** *Component-level first-event summary for the primary composite endpoint*

| **Patient No.** | **SARS-CoV-2 infection date** | **First counted event component** | **First event date** | **Additional or overlapping component(s)** | **Endpoint interpretation** |
| --- | --- | --- | --- | --- | --- |
| 2 | 13 Jan 2022 | PH clinical deterioration requiring escalation of therapy | 12 Sep 2022 | No additional death, lung transplantation, or ICU component recorded | Treprostinil pump insertion/initiation was counted as PH deterioration requiring therapy escalation. |
| 5 | 18 Apr 2022 | ICU admission with same-date death | 4 May 2022 | Death due to right-heart failure recorded on the same date | Counted once as the first composite event; ICU retained as a descriptive ICU component and death retained descriptively. |
| 11 | 27 Jan 2022 | All-cause death | 8 Feb 2023 | No ICU admission or lung transplantation component recorded | Death was the first documented qualifying composite endpoint component. |
| 12 | 23 Jan 2022 | PH clinical deterioration requiring escalation of therapy | 6 Nov 2022 | IV treprostinil admission and later lung transplantation on 8 May 2023 | PH deterioration was the first qualifying component; lung transplantation was retained as a subsequent descriptive component. |
| 13 | 29 Aug 2021 | ICU admission | 11 Aug 2022 | Death due to right-heart failure recorded on 8 Dec 2022 | ICU admission was the first documented qualifying component and was retained as one of the two manuscript-level ICU components. |
| 26 | 12 Sep 2021 | All-cause death | 15 Sep 2021 | ICU-level care and PH deterioration recorded on the same date as death | The terminal same-date episode was counted as a patient-level death event; the raw ICU-level record was retained descriptively but not counted as an independent manuscript-level ICU component. |
| 35 | 16 Sep 2020 | PH clinical deterioration requiring hospitalization | 11 May 2022 | No death, lung transplantation, or ICU component recorded | Post-infection PH deterioration was the first documented qualifying composite endpoint component. |

*The primary composite endpoint was analysed as time to first qualifying event at the patient level. Patients with overlapping or sequential endpoint components contributed only the first chronological event to the survival analysis; later or overlapping components were retained descriptively. PH denotes pulmonary hypertension; ICU denotes intensive care unit; IV denotes intravenous.*
