# Supplementary material for: SARS-CoV-2 infection and two-year longitudinal clinical trajectory in pulmonary hypertension: a single-center retrospective exploratory cohort study
Source: Front Public Health. 2026 Sep 24;14:1901917. doi: 10.3389/fpubh.2026.1901917 (PMC13646160; doi:10.3389/fpubh.2026.1901917)
Supplement: Supplementary file 3 [file Table_3.docx]

***Supplementary Table S3.*** *Exploratory linear mixed-effects sensitivity analyses for continuous longitudinal outcomes — expanded estimates with 95% confidence intervals*

| **Outcome / transformation** | **Records / patients** | **Contrast** | **Model-based change versus T0 (95% CI)** | **Overall time-window p value** | **Interpretive scale** |
| --- | --- | --- | --- | --- | --- |
| Haemoglobin (no transformation) | 236 records / 40 patients | T1 vs T0 | −3.8 g/L (−10.1 to 2.4) | 0.21 | Mean difference (g/L) |
|  |  | T2 vs T0 | −4.3 g/L (−10.0 to 1.4) |  |  |
|  |  | T3 vs T0 | −1.5 g/L (−7.4 to 4.4) |  |  |
|  |  | T4 vs T0 | +4.6 g/L (−4.3 to 13.6) |  |  |
| NT-proBNP (log10-transformed) | 230 records / 40 patients | T1 vs T0 | 1.22 (0.93 to 1.61) | 0.72 | Geometric-mean ratio |
|  |  | T2 vs T0 | 1.06 (0.83 to 1.37) |  |  |
|  |  | T3 vs T0 | 1.07 (0.82 to 1.39) |  |  |
|  |  | T4 vs T0 | 1.09 (0.73 to 1.62) |  |  |
| 6MWD (no transformation) | 177 records / 39 patients | T1 vs T0 | −9 m (−39 to 21) | 0.67 | Mean difference (m) |
|  |  | T2 vs T0 | −5 m (−29 to 20) |  |  |
|  |  | T3 vs T0 | +6 m (−19 to 32) |  |  |
|  |  | T4 vs T0 | +17 m (−21 to 55) |  |  |

*Values are from patient-level linear mixed-effects models fitted by maximum likelihood with follow-up window as a categorical fixed effect and a patient-specific random intercept. T0 was the reference category. Overall p values are likelihood-ratio tests comparing models with and without the follow-up-window term. Contrast intervals are two-sided 95% Wald confidence intervals. For haemoglobin and 6MWD, effects are mean differences versus T0; for NT-proBNP, log10-scale coefficients were back-transformed and are presented as geometric-mean ratios versus T0. The longitudinal windows used for model assignment were T1 <90 days, T2 90–364 days, T3 365–729 days, and T4 ≥730 days after the index infection date. Confidence intervals were not adjusted for multiplicity because these analyses were exploratory sensitivity analyses. The intervals for all T1–T4 contrasts included the null value (0 for mean differences; 1 for ratios).*
