# Supplementary material for: SARS-CoV-2 infection and two-year longitudinal clinical trajectory in pulmonary hypertension: a single-center retrospective exploratory cohort study
Source: Front Public Health. 2026 Sep 24;14:1901917. doi: 10.3389/fpubh.2026.1901917 (PMC13646160; doi:10.3389/fpubh.2026.1901917)
Supplement: Supplementary file 4 [file Table_4.docx]

***Supplementary Table S4.*** *Contextual variables relevant to confounding and interpretation*

| Contextual variable | Information available for analysis | Interpretive consideration |
| --- | --- | --- |
| SARS-CoV-2 vaccination status | Vaccination information was not available as a structured analysis variable. | Potential modification of acute infection severity or subsequent trajectory by vaccination could not be evaluated. |
| SARS-CoV-2 variant context | Individual viral lineage data were not available. Confirmed infections occurred between 15 June 2020 and 2 November 2022. | Calendar-time and variant-related heterogeneity may have influenced clinical course, but variant-specific effects were not assessed at the patient level. |
| Acute COVID-19 care setting | Acute/index care setting was available descriptively. At T0, 38 patients were managed as outpatients, two required conventional-ward care, and no ICU admission was recorded in the available-record summary. | Care setting provides a descriptive proxy for acute COVID-19 severity, but a formal severity score was not available for modelling. |
| PH treatment escalation | Therapy escalation was captured when documented as part of PH clinical deterioration or endpoint ascertainment. Systematic time-varying medication data for all patients were not available in the analysis dataset. | Treatment changes may reflect or modify disease trajectory and were interpreted descriptively rather than as adjustment covariates. |
| Comorbidity profile | PH phenotype, demographic variables, and selected clinical variables were available. A comprehensive non-PH comorbidity index was not available as a structured variable. | Residual confounding by systemic comorbidity burden cannot be excluded. |
| SARS-CoV-2 reinfection | The analysis was anchored to the first documented positive SARS-CoV-2 RT-PCR test. Subsequent SARS-CoV-2 infections were not available as a structured longitudinal exposure. | Potential effects of reinfection on later follow-up windows could not be formally evaluated. |
| Concurrent respiratory infection | Concurrent respiratory infections were not consistently available as structured variables; isolated infectious events may have been present in narrative clinical records. | Their contribution to longitudinal clinical changes could not be modelled separately. |
| Pre-infection PH stability | A formal pre-infection stability index or standardized pre-infection control period was not available. | Underlying pre-infection trajectory may have contributed to post-infection changes; causal interpretation should therefore remain cautious. |

*This table summarises the availability of contextual variables relevant to residual confounding and interpretation of the post-SARS-CoV-2 PH trajectory. Availability refers to variables captured in a form suitable for descriptive analysis or formal modelling in the retrospective analysis dataset and does not imply that unavailable variables were clinically absent. RT-PCR denotes reverse-transcription polymerase chain reaction; PH denotes pulmonary hypertension; ICU denotes intensive care unit.*
