# Supplementary material for: SARS-CoV-2 infection and two-year longitudinal clinical trajectory in pulmonary hypertension: a single-center retrospective exploratory cohort study
Source: Front Public Health. 2026 Sep 24;14:1901917. doi: 10.3389/fpubh.2026.1901917 (PMC13646160; doi:10.3389/fpubh.2026.1901917)
Supplement: Supplementary file 5 [file Table_5.docx]

***Supplementary Table S5.*** *STROBE reporting checklist for observational cohort studies*

| STROBE item | Recommendation | Manuscript Segment |
| --- | --- | --- |
| Title and abstract 1a | Indicate the study design with a commonly used term in the title or abstract. | Title; Abstract |
| Title and abstract 1b | Provide an informative and balanced summary of what was done and what was found. | Abstract |
| Background/rationale 2 | Explain the scientific background and rationale for the investigation. | Introduction |
| Objectives 3 | State specific objectives, including any prespecified hypotheses. | Introduction |
| Study design 4 | Present key elements of the study design early in the manuscript. | Materials and Methods |
| Setting 5 | Describe the setting, location, and relevant dates, including recruitment, exposure, follow-up, and data collection periods. | Materials and Methods; Results |
| Participants 6a | Give eligibility criteria, sources and methods of participant selection, and methods of follow-up. | Materials and Methods |
| Participants 6b | For matched studies, give matching criteria and the number of exposed and unexposed participants. | Not applicable; the study did not use a matched exposed/unexposed cohort design. |
| Variables 7 | Define outcomes, exposures, predictors, potential confounders, and diagnostic criteria, as applicable. | Materials and Methods |
| Data sources/measurement 8 | For each variable of interest, give data sources and details of assessment or measurement methods. | Materials and Methods |
| Bias 9 | Describe efforts to address potential sources of bias. | Materials and Methods: Statistical analysis; Results; Discussion: Limitations |
| Study size 10 | Explain how the study size was arrived at. | Materials and Methods; Results; Figure 1 |
| Quantitative variables 11 | Explain how quantitative variables were handled in the analyses, including any categorizations. | Materials and Methods: Statistical analysis; Table 2; Table 3 |
| Statistical methods 12a | Describe all statistical methods, including methods used to address confounding. | Materials and Methods: Statistical analysis; Results |
| Statistical methods 12b | Describe methods used to examine subgroups and interactions. | Results; Discussion: Limitations |
| Statistical methods 12c | Explain how missing data were addressed. | Materials and Methods; Results; Table 2; Discussion: Limitations |
| Statistical methods 12d | If applicable, explain how loss to follow-up was addressed. | Materials and Methods; Table 2 |
| Statistical methods 12e | Describe sensitivity analyses. | Materials and Methods; Results; Supplementary Tables S1-S3 |
| Participants 13a | Report the number of individuals at each stage of the study. | Results; Figure 1 |
| Participants 13b | Give reasons for non-participation or exclusion at each stage. | Materials and Methods; Results; Figure 1 |
| Participants 13c | Consider use of a flow diagram. | Figure 1 |
| Descriptive data 14a | Give characteristics of study participants and information on exposures and potential confounders. | Table 1; Table 2; Supplementary Table S4 |
| Descriptive data 14b | Indicate the number of participants with missing data for each variable of interest. | Materials and Methods; Results; Table 2 |
| Descriptive data 14c | Summarise follow-up time. | Materials and Methods; Results; Table 2 |
| Outcome data 15 | Report numbers of outcome events or summary measures over time. | Results; Supplementary Table S2 |
| Main results 16a | Give unadjusted and, if applicable, adjusted estimates with precision; specify variables adjusted for and why they were included. | Materials and Methods; Results; Table 3 |
| Main results 16b | Report category boundaries when continuous variables were categorized. | Materials and Methods; Table 3 |
| Main results 16c | If relevant, translate relative-risk estimates into absolute risk for a meaningful time period. | Results; Figure 3 |
| Other analyses 17 | Report other analyses, such as subgroup, interaction, and sensitivity analyses. | Results; Supplementary Tables S1-S3 |
| Key results 18 | Summarise key results with reference to study objectives. | Discussion |
| Limitations 19 | Discuss limitations, including sources of potential bias or imprecision and their possible influence. | Discussion: Limitations |
| Interpretation 20 | Provide a cautious overall interpretation considering objectives, limitations, multiplicity of analyses, similar studies, and other evidence. | Discussion; Conclusions |
| Generalisability 21 | Discuss the generalisability or external validity of the study results. | Discussion: Limitations; Conclusions |
| Funding 22 | Give the source of funding and the role of funders for the present study and, if applicable, the original study on which the article is based. | Funding statement |

*STROBE denotes Strengthening the Reporting of Observational Studies in Epidemiology. Section, subsection, table, and figure references correspond to the revised manuscript submitted with this supplementary material. The checklist is based on the STROBE Statement recommendations for reporting cohort studies.*
